# Analyses of the sucrose synthase gene family in cotton: structure, phylogeny and expression patterns

**DOI:** 10.1186/1471-2229-12-85

**Published:** 2012-06-13

**Authors:** Aiqun Chen, Shae He, Feifei Li, Zhao Li, Mingquan Ding, Qingpo Liu, Junkang Rong

**Affiliations:** 1 School of Agriculture and Food Science, Zhejiang A & F University, Lin'an, Hangzhou, Zhejiang 311300, China; 2 State Key Laboratory of Crop Genetics and Germplasm Enhancement, College of Resources and Environmental Sciences, Nanjing Agricultural University, Nanjing 210095, China

**Keywords:** Sucrose synthase, Gene structure, Phylogeny, Expression pattern, Cotton

## Abstract

**Background:**

In plants, sucrose synthase (Sus) is widely considered as a key enzyme involved in sucrose metabolism. Several paralogous genes encoding different isozymes of Sus have been identified and characterized in multiple plant genomes, while limited information of Sus genes is available to date for cotton.

**Results:**

Here, we report the molecular cloning, structural organization, phylogenetic evolution and expression profiles of seven Sus genes (*GaSus1* to 7) identified from diploid fiber cotton (*Gossypium arboreum*). Comparisons between cDNA and genomic sequences revealed that the cotton *GaSus* genes were interrupted by multiple introns. Comparative screening of introns in homologous genes demonstrated that the number and position of Sus introns are highly conserved among Sus genes in cotton and other more distantly related plant species. Phylogenetic analysis showed that *GaSus1*, *GaSus2*, *GaSus3*, *GaSus4* and *GaSus5* could be clustered together into a dicot Sus group, while *GaSus6* and *GaSus7* were separated evenly into other two groups, with members from both dicot and monocot species. Expression profiles analyses of the seven Sus genes indicated that except *GaSus2*, of which the transcripts was undetectable in all tissues examined, and *GaSus7*, which was only expressed in stem and petal, the other five paralogues were differentially expressed in a wide ranges of tissues, and showed development-dependent expression profiles in cotton fiber cells.

**Conclusions:**

This is a comprehensive study of the Sus gene family in cotton plant. The results presented in this work provide new insights into the evolutionary conservation and sub-functional divergence of the cotton Sus gene family in response to cotton fiber growth and development.

## Background

Sucrose is the major form of photosynthate for export from the source leaves into cellular metabolism of most plants [1]. The transfer of sucrose to the sink organs often requires its cleavage by two key enzymes: invertase (Inv), which hydrolyzes sucrose into glucose and fructose, and sucrose synthase (Sus), which is capable of catalyzing a reversible reaction but preferring to convert sucrose and UDP into fructose and UDP-glucose [2-4]. Both of the two enzymes have been demonstrated to be tightly linked with the processes of phloem unloading [5,6], and the Sus has also been well characterized in various plants as playing crucial roles in regulation of carbon partitioning into various pathways that important for storage functions and metabolic structure of the plant cell [7,8]. For instance, Sus cleavage activity has been documented repeatedly to be highly correlated with sink strength of various starch storing organs including potato tubers, carrot roots, maize kernels and pea embryos [9-13]. Sus activity is also proposed to be responsible for cellulose synthesis, by supplying UDP-glucose as substrates, which has been shown to be essential for cell wall thickening and cotton fiber cell development [14-16]. In addition, Sus activity is also considered to be associated with other important metabolic processes such as sugar import [17,18], environmental stresses response [19,20], and nitrogen fixation as well as arbuscule maturation and maintenance in mycorrhizal roots of legumes [21,22].

The identification and subsequent characterization of the genes encoding plant sucrose synthase is the first step towards understanding their physiological roles and metabolic mechanism involved in different growth processes. Recent studies have revealed that Sus isozymes are encoded by a small, multigene family that comprises at least three Sus genes in the most plant species. With the completion of the genome sequence analysis, recent studies showed that model plant species, *Arabidopsis* and rice, both contain an Sus gene family with six distinct active Sus genes, suggesting gene expansion in higher plants during evolution [23,24]. Similarly in the model legume *Lotus japonicus*, at least six Sus genes are known to exist in the Sus gene family [25]*.* Studies on these Sus sequences and phylogenetic relatedness revealed that structural conservation and functional divergence occurred within the gene family during evolution. Members of the Sus gene family in many plant species are divergent in function and differentially expressed during plant development. For example, maize harbors a Sus family containing at least three distinct genes, *Sh1Sus1* and *Sus3*. *Sh1* is most abundantly expressed in developing endosperm, and has the dominant role in cell wall synthesis. *Sus1* is expressed in a wide range of tissues, but plays a major role in starch synthesis [26,27]. The pea Sus gene family also includes at least three divergent members, *Sus1Sus2* and *Sus3. Sus1* is ubiquitously and highly expressed in the developing seed. *Sus2* is mainly expressed in older testas and leaves, while *Sus3* is weakly expressed only in flowers and young testas. Furthermore, the lack of *Sus1* activity in mutant seeds and root nodules could not be compensated by *Sus2* and *Sus3*[13]. *Arabidopsis* contains three major Sus gene classes with distinct but partially overlapping expression profiles, and the specific roles have been assigned for each gene by extensive studies of corresponding knockout mutants [20]. In other plant species, such as rice, *Lotus japonicus* and citrus, Sus genes have also been demonstrated to be expressed in tissue-specific and development-dependent patterns [24,25,28,29]. In all cases, the differential expression of Sus genes implies that each Sus isoform may have evolved into specialized functions in different tissues. Although Sus genes in a few plant species such as *Arabidopsis*, have been well studied, our knowledge of cotton Sus genes, especially their evolutionary mechanisms and potential functions in fiber growth and development, needs to be well explored.

Cotton fiber is not only the world's most important textile material, but also the ideal experimental system for studying the mechanism of cell development based on its single-celled profile [30]. Cotton fiber is originated from epidermal cells of ovules, and its growth and development is a highly gene-regulated process involving four distinct, but overlapping stages: initiation, elongation, secondary wall synthesis, and maturation [31]. Many genes, including the Sus genes, have been proposed to be involved in controlling cotton fiber development [32]. Previous studies based on southern blot analysis suggested the existence of a small gene family encoding different Sus isoforms within the tetraploid upland cotton genome [33], but to date limited works to characterize these particular Sus genes have been reported, except for *SS3* (*GhSus3*, accession no. U73588), which was isolated previously from an upland cotton fiber cDNA library and has been demonstrated to play an important role in ovule development and fiber cell initiation [34]. For the purpose of gaining a comprehensive understanding of the molecular and evolutionary characterization as well as the possible functions of cotton Sus family, it is of great necessity and significance to identify and subsequently determine the expression patterns of any members belonging to this gene family.

In the current work, we reported the identification and characterization of seven Sus genes in cotton species and investigated their expression patterns at the transcriptional level. The analysis in this study mainly focused on the gene identification, evolutionary relationship, exon/intron organization and tissue-specific expression patterns of each member of the cotton Sus gene family. Therefore, our results obtained from this study will provide a foundation and framework for the further studies to gain a comprehensive understanding of the physiological roles of each cotton Sus gene in regulating the cotton plant growth, especially for the growth and development of cotton fiber.

## Results

### Cloning and sequence analysis of Sus cDNAs in diploid fiber cotton

In order to identify the potential Sus homologues in cotton, comparisons between coding sequences of plant known Sus genes were performed and regions exhibiting somewhat similarities were picked out and used to design multiple pairs of degenerate primers for PCR amplification. The genomic DNA of diploid fiber cotton (*G. arboreum*), rather than the allotetraploid cotton species, was used as a template, to avoid the laborious analysis of allelic sequence variation within the A_T_ and D_T_ sub-genomes. Eventually, five distinct non-allelic genomic fragments exhibiting substantial homology to plant Sus genes were identified by massive sequencing of the PCR-generated clones. In addition, using the mRNA sequences of *Arabidopsis* Sus genes as queries, the cotton EST database was extensively searched, leading to the assembly of other two additional contigs as putative Sus genes. The seven newly isolated gene fragments were named as *GaSus1* to *7*, respectively, according to the naming convention for Sus genes in other plant species. 5' and 3' RACE subsequently resulted in the cloning of the full-length cDNAs of these genes, except *GaSus2*, of which the mRNA could not be detectable in all tissues of cotton (described later).

**Figure 1:**
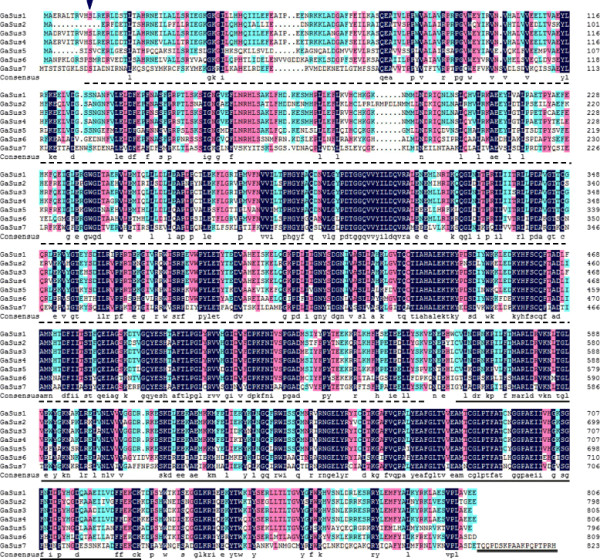
**Predicted amino acid sequences of the seven cotton Sus genes *GaSus1-7 *(accession no. *GaSus1*, JQ995522; *GaSus2*, JQ995523; *GaSus3*, JQ995524; *GaSus4*, JQ995525; *GaSus5*, JQ995526; *GaSus6*, JQ995527; *GaSus7*, JQ995528).** Sequence alignment analysis was performed using Multiple alignment program wrapped within the DNAMAN 6.0 software ( http://www.lynnon.com/). Identical amino acids are shaded and gaps are indicated by dots. The conserved serine residue for phosphorylation by Ser/Thr protein kinase is showed by an arrowhead. The characteristic sucrose synthase domain (broken underline) and a glycosyl transferases domain (single underline) were identified by the Interproscan algorithm ( http://www.ebi.ac.uk/Tools/pfa/iprscan/). The extended C-terminal region of *GaSus7* is shown by double underlines.

Molecular analysis of the full-length deduced polypeptides indicated that the putative proteins of these cotton Sus genes contain 796–824 amino acids (predicted 90.26-93.14 kDa in molecular weight) with their isoelectric point calculated to lie between 6.26 and 7.27 (Additional File 1), similar to the molecular feature of Sus isozymes from other plant species. Additionally, all the GaSus amino acid sequences, except GaSus2, share the conserved Ser residue in the N-terminal regions (Figure 1), which has been documented to be phosphorylated by the Ser/Thr protein kinase in maize [35,36]. Furthermore, using the Interproscan algorithm ( http://www.ebi.ac.uk/interpro/), two conserved, sucrose synthase and the glucosyl-transferase domains, which have been suggested to be typical signatures of Sus proteins were also identified in all the cotton *GaSus* genes (Figure 1). These findings led to the suggestion that these newly isolated genes encode different isozymes of sucrose synthase in cotton.

Multiple sequence alignment using the DNAMAN algorithm revealed high levels of similarities between the coding sequences (CDSs) of *GaSus1* to *5*, within which *GaSus1*, *GaSus3* and *GaSus4* were found to be the more closely related genes, sharing much higher sequence identities (85.4-87.8% at the nucleotide level, and 94.3-94.9% at the amino acid level), compared to the other paralogues (Table 1). Moreover, except *GaSus3* had a Val/Glu deletion at the third last position of the C termini, there is only 3% substitution rate at amino acid level between the three proteins compared with each other (Additional File 2). The other two paralogues, *GaSus6* and *GaSus7*, had relatively lower levels of identities when compared to *GaSus1* to *5*, or compared with each another (Table 1).

**Table 1:** **Identity matrix for the seven*GaSus*coding sequences and their predicted amino acid sequences**

		**Amino acid identity**						
		**GaSus1**	**GaSus2**	**GaSus3**	**GaSus4**	**GaSus5**	**GaSus6**	**GaSus7**
Nucleotide identity	GaSus1	-	86.1	94.9	94.4	77.4	71.2	55.7
	GaSus2	83.1	-	86.2	86.7	77.0	68.3	57.0
	GaSus3	87.1	83.4	-	94.3	77.1	71.6	55.0
	GaSus4	85.4	83.9	87.8	-	76.6	70.8	55.2
	GaSus5	74.8	75.0	75.2	74.5	-	64.3	55.1
	GaSus6	67.7	66.9	68.5	68.5	64.7	-	56.0
	GaSus7	58.5	59.8	59.2	58.7	59.0	59.5	-

### Exon/Intron organization of the cotton Sus gene family

**Figure 2:**
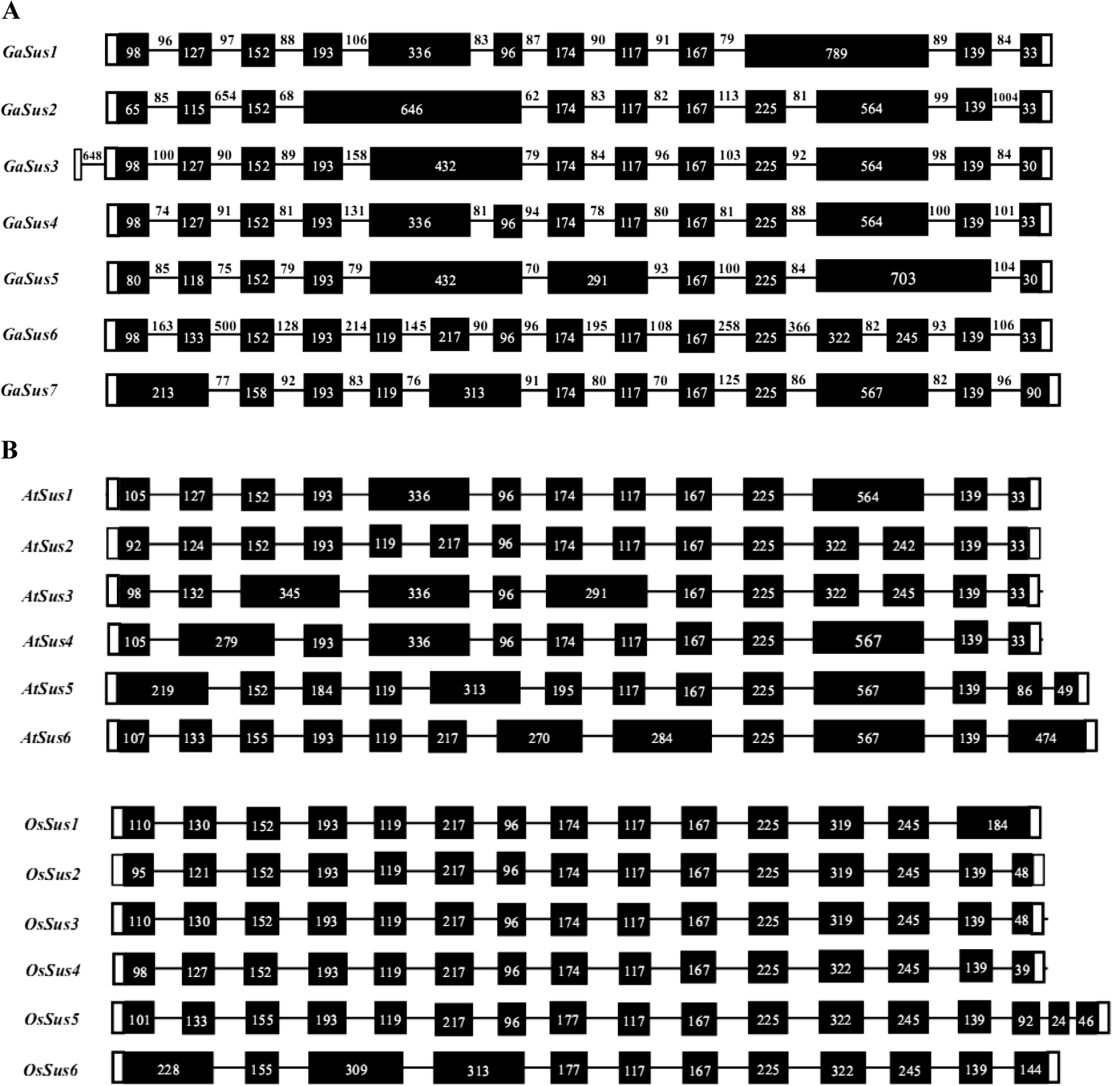
**Exon/Intron structural organization of plant Sus genes. (A)** Exon/intron structures of the seven sucrose synthase genes, *GaSus1* to *7*, from diploid fiber cotton (*G. Arboreum*, A2). Black boxes denote exons within coding regions, and the lines connecting them represent introns. Numbers in boxes or above lines represent the sizes (bp) of corresponding exons or introns, respectively. The 5' and 3' untranslated regions (UTRs) are represented by blank boxes. For highlighting the conservation of exon structures, the sizes of exons, introns and non-coding UTRs are not actually presented. **(B)** Exon/intron structures of the sucrose synthase genes, *AtSus1-6* and *OsSus1-6*, from *Arabidopsis* and rice, respectively.

Comparative analysis of the exon/intron gene structure may provide some clues for the understanding of the evolutionary mechanisms underlying the genesis of family genes. In order to investigate the exon/intron structure of the cotton *GaSus* genes, seven genomic DNA sequences, encompassing putative overall coding regions of *GaSus1* to *7*, were cloned from the *G. arboreum* genome. A comparison between the cDNA (except *GaSus2*) and genomic sequences, revealed that these *GaSus* genes are interrupted by multiple introns. As shown in Figure 2a (to highlight the exon structures, the size of exons, introns and non-coding regions were not actually presented), *GaSus1*, *GaSus3* and *GaSus7* all contain 11 introns in their coding regions (between the start and stop codons), while *GaSus4* and *GaSus6* are characterized by one and three more introns (12 and 14 introns, respectively) than the three paralogues. However, *GaSus5* is characterized by having only 9 introns. As the absence of the cDNA sequence, the exon/intron structure of *GaSus2* was predicted online using the FGENESH algorithm ( http://linux1.softberry.com/berry.phtml?topic=fgenesh&group=programs&subgroup=gfind), which resulted in the definition of 10 putative introns within its putative coding regions. The last intron, with a length more than one kb, was predicted to be the largest intron located in the cotton Sus gene family. Comparative analysis of these introns indicated a marked greater difference in sequence similarities compared to that shown by the exons in *GaSus* genes (data not shown). In addition, most of the intron sizes of the seven *GaSus* genes range within 70–110 bp in length, except *GaSus6*, within which 57% (8 of 14) introns are larger than 120 bp in length (Figure 2a). Interestingly, the third largest intron (648 bp) was found to locate in the 5' untranslated regions (UTR) of *GaSus3*, and was very close (9 bases) to the ATG start codon. In addition, all the introns confer the GT-AG splicing rule, except for the sixth intron of *GaSus7*, which was spliced at the unusual GC-AG splicing sites.

**Figure 3:**
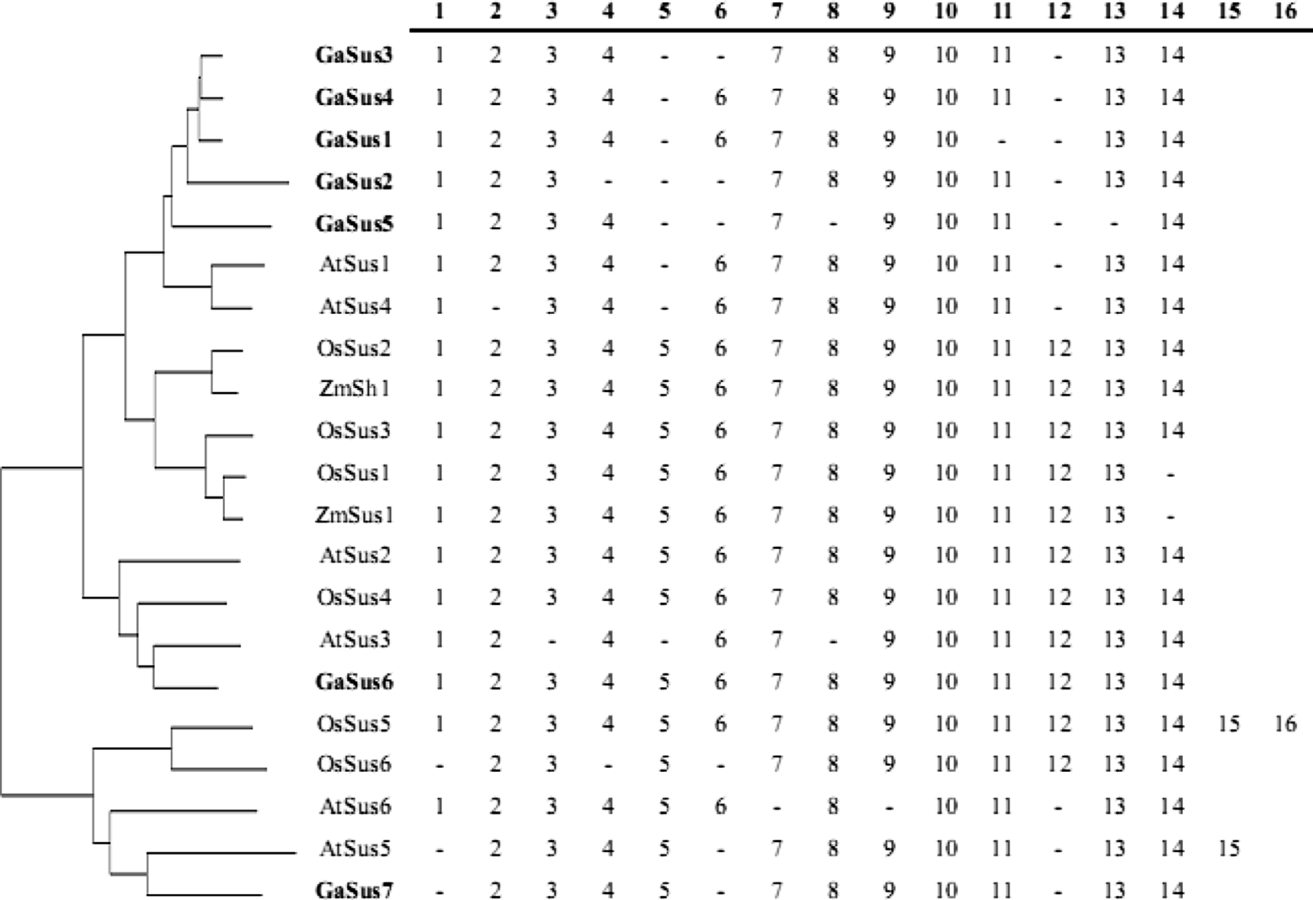
**Schematic presentation of the conservation of intron numbers and positions of Sus genes from four phylogenetically distant plant species: cotton, *Arabidopsis, *rice and maize.** Sequences are ordered according to the phylogenetic relationship of the corresponding coding regions, as shown on the left. Intron positions are indicated in relation to amino acid sequence of the position of exons. Dashes indicate the loss of introns in corresponding positions.

Similar to the high identities shown by the alignment of amino acid sequence, high conservation of exon/intron structures could also been observed from the schematic representation of cotton Sus genes. As observed in Figure 2b, most of the introns are shared (located in the same position related to the position of exons) between the Sus homologues not only in cotton, and also in the phylogenetically distant species *Arabidopsis* and rice. Moreover, *GaSus7* exhibited an extension of the last exon compared to the other *GaSus* genes, such feature are also observed, but more conspicuous in the *Arabidopsis AtSus6* and the rice *OsSus6* orthologues. Alignments of both the cDNA sequences and their predicted amino acid sequences between *GaSus7*, *AtSus6* and *OsSus6* revealed poor identities in the extended regions of the last exons, but much high identities in the other coding sequence (data not shown)*.* Moreover, similarity searches with the BLAST algorithm against the NCBI database using the extended regions of the three orthologues failed to hit any sequence, except themselves, that showed significant similarities. In addition, careful scrutiny of the exon/intron structure in coding regions of Sus genes from two dicots (cotton and *Arabidopsis*) and two monocots (rice and maize) revealed that among these 21 sequences, introns were present at a total of 16 positions, in which 14 introns, in conserved positions, were observed to be contained in most of the Sus sequences from the four distantly related species, suggesting that ancestral Sus genes common to monocots and dicots contained 14 introns in these positions. The presence of an intron in the 5' untranslated region of cotton *GaSus3* sequence and introns 15 and 16 at novel positions in the *Arabidopsis AtSus5* and rice *OsSus5* sequences may represent derived characters during evolution. Several of the Sus genes lack one or more introns (Figure 2a,b and Figure 3), led to the suggestion that this occurred by intron loss. Interestingly, intron loss events occurred mainly associated with the fifth, sixth and 12th introns of dicots Sus genes, resulting in the formation of relatively larger exons, such as 336, 432 and 564(567) bp- length exons in some cotton and *Arabidopsis* homologues.

To further investigate evolutionary conservation and sequence divergence of Sus genes among different cotton species, we also cloned the orthologues of the seven *GaSus* genes from other three wild diploid cotton genomes: *G. anomalum* (B_1_), *G. sturtianum* (C_1_) and *G. raimondii* (D_5_), as well as from an outgroup species *Gossyploides kirkii* (K). Sequence comparison and gene structure prediction of the orthologous genes from the other four diploid cotton relatives revealed identical amino acid sizes (Additional File 1), consistent exon/intron structures and very high simialities not only in coding sequences, but also in noncoding regions to the corresponding orthologs in *G. arboreum* (Additional File 3). Moreover, comparisons between the Sus gene sequences in the five cotton diploid species permitted the identification of several SSRs (simple sequence repeats; microsatellites) located within different introns of the orthologues of seven Sus genes (Additional File 3), which might be further used to develop SSR or intronic polymorphism markers in cotton lineages.

### Phylogenetic analysis of cotton Sus genes and other plant Sus homologs

**Figure 4:**
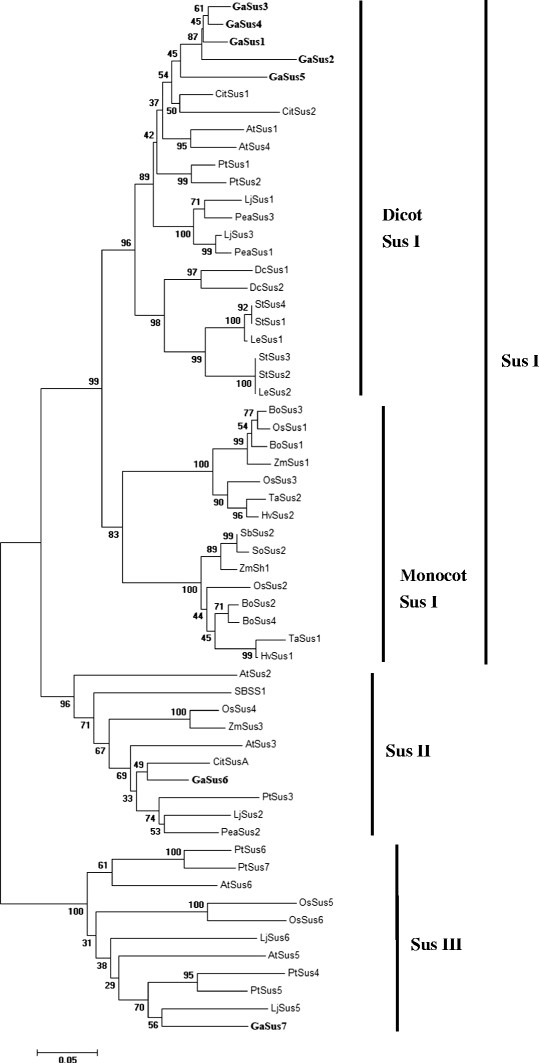
**Phylogenetic analysis of cotton Sus genes and other plant Sus homologs.** Unrooted phylogenetic tree of plant Sus proteins constructed using the neighbor-joining method with MEGA 5.0 program. Isozymes and corresponding plant species are: cotton, GaSus1 to 7 (this study); Potato, StSus1 to 4 [9]; Pea, PeaSus1 to 3 [13]; Wheat, TaSus1 and TaSus2 [15]; *Arabidopsis thaliana*, AtSus1 to 6 [23]; Rice, OsSus1 to 6 [24]; *Lotus japonicus*, LjSus1 to 6 [25]; Citrus, CitSus1, CitSus2 and CitSusA [29]; Bamboo, BoSus1 to 4 [37]; Tomato, LeSus1 and LeSus2 [38]; Carrot, DcSus1 and DcSus2 [39]; Maize, ZmSh1, ZmSus1 and ZmSus3 [40]; Barley, HvSus1 and HvSus2 [41]; Sugarbeet, SbSS1 [42]; Sorghum, SbSus2 (accession no. FJ513325); Sugarcane, SoSus2 (accession no. AY118266); Populus, PtSus1 to 7 [43].

In order to carry out a comprehensive analysis of evolutionary relationships among Sus gene families between cotton and other plant species, including the seven isoforms of cotton Sus, a total of 59 plant Sus amino acid sequences, representing 17 species, were aligned with the ClustalX program and used to construct an unrooted tree for phylogenetic analysis using Neighbour-Joining method. We also performed a bootstrap analysis (1000 replicates) to determine the robustness of the phylogram's topology. The phylogenetic tree analysis revealed both relatively deep evolutionary root and the existence of more recent duplications for the Sus genes. As shown in Figure 4, three major groups of Sus proteins are inferred (for simplicity in this paper, the three groups were named as Sus I, II and III, respectively), as they are supported by high bootstrap values >95%. Genes from dicot- and monocotyledonous plants are found in all the three groups, suggesting their evolutionary divergence before the common ancestor of dicots and monocots. In addition, Sus proteins in Sus I group can be well classified into two distinct subclades, consisting of one dicot-specific Sus I group and one monocot-specific Sus I group.

The dicot Sus I group contains the proteins solely from the dicotyledonous plant species, in which they are subgrouped by phylogeny. For example, one group of solanaceous Sus proteins, including members from tomato and potato species, forms a cluster together with the two Daucus carrot proteins, DcSus1 and DcSus2. These proteins are part of the largest group that includes brassicaceous, daucus, solanaceous, leguminous, populus and citrus proteins but not a single cotton proteins. For cotton, five of the seven *GaSus* genes (*GaSus1* to *5*) fallen into this dicot group, and cluster together by forming an independent cotton clade to the exclusion of citrus, *Arabidopsis*, as well as other dicots genes, suggesting that a single Sus gene has expanded through independent duplication within the cotton lineages occurred after cotton separation with citrus and *Arabidopsis* species. Within the cotton clade, three paralogues, *GaSus1, GaSus3* and *GaSus4,* which share a very high degree of sequence similarities, grouped more closely in the phylogenetic tree. The short branches separating the three genes suggesting that duplications that gave rise to them occurred relatively recently. In addition, *GaSus5* was found to be unambiguously distant from the other four paralogues within this group. In contrast to dicot Sus I group, the monocot Sus I group thus includes members exclusively from monocot species, in which the proteins can be divided into two subclasses. Both of the subclasses contain Sus genes from rice, maize, wheat, barley and bamboo, suggesting that some ancestor Sus genes had diverged before gramineous species evolved into different plant lineages, and then underwent independent evolution within each lineage.

Unlike cotton Sus genes expanded in dicot Sus I group, the other two paralogues, *GaSus6* and *GaSus7*, were evenly separated into the other two groups, Sus II and III, with members from both dicot and monocot species. Additionally, in these two groups, genes from dicots and monocots were not found in distinct subgroups, suggesting that the significant duplications that gave rise to Sus II and III groups should be no younger than the monocots/dicots split.

### Expression analysis of the cotton Sus genes at different developmental stages

**Figure 5:**
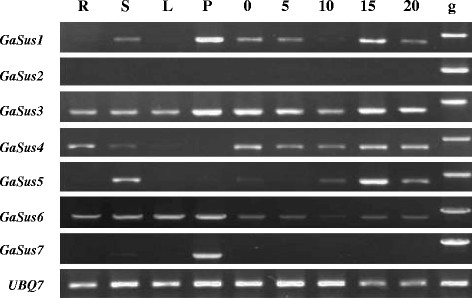
**Semiquantitative RT-PCR analysis of the seven *GaSus *genes in cotton plants (*G. arboreum, A2*).** The RNA were prepared from different tissues and over fiber development: root (R), stem (S), young leaf (L), petal (P), ovule at 0 dpa (day post anthesis) and fiber samples from bolls at 5, 10, 15, and 20 dpa. Primers specific to the seven *GaSus* were used, and the PCR products amplified from genomic DNA (g) containing at least one introns were taken as controls to exclude any DNA contamination. A constitutive ubiquitin gene (*UBQ7*, accession no. DQ116441) was used as an internal control to normalize differences in template concentrations.

Systematic analysis of the expression patterns of Sus family genes can help to reveal their possible physiological functions involved in different growth processes in plant. In order to better learn the potential functions of specific isoforms of Sus in cotton, the tissue-specific expression of *GaSus* genes were firstly examined in various cotton tissues, including roots, stems, petals and young leaves, as well as fibers at different developmental stages using semi-quantitative RT-PCR. It is worth to point out that there is a very high sequence similarity within the encoding sequence of some *GaSus* genes, and their reverse primers were therefore designed within their 3' untranslated regions to guarantee the primer specificity. As shown in Figure 5, except *GaSus2*, of which the transcripts was undetectable in all tissues examined, and *GaSus7*, of which the transcripts could only be detected in stem and petal, the other *GaSus* transcripts were detected in a wide range of tissues and showed distinct but partially overlapping expression patterns, suggesting that Sus genes might be implicated in a range of physiological processes in cotton plant.

**Figure 6:**
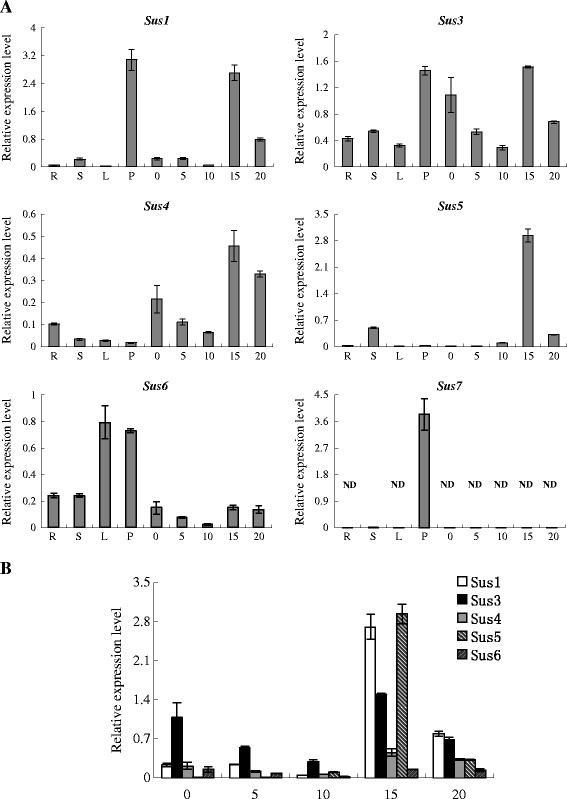
**Real-time RT-PCR analysis of *GaSus *genes in different cotton tissues. (A)** Relative value of *GaSus* gene expression in cotton tissues, including root (R), stem (S), young leaves (L), petal (P), ovule at 0 dpa (day post anthesis) and fiber samples from bolls at 5, 10, 15, and 20 dpa, was shown as percentage of *GaUBQ7* expression activity (see Methods). ND indicates that transcripts were not detectable. **(B)** Relative expression level of five fiber-developmental related cotton *GaSus* genes at different fiber developmental stages.

Real-time RT-PCR was further performed to quantitatively determine the relative expression level of specific *GaSus* gene in different tissues and in fibers at different developmental stages (Figure 6a). The quantitative results showed a similar expression profiles to those observed from semi-quantitative RT-RCR. In detail, *GaSus1* was expressed highly in petal and fiber at 15 dpa, and also to some extent in stem, ovules and fibers at other developmental stages, while very slightly in roots and leaves. *GaSus3* was ubiquitously expressed in all tissues, and the transcription levels were highly detected in petal, ovule at 0 dpa and fiber at 15 dpa. In addition, the transcripts of *GaSus3* in fiber were decreased as the fiber development from 0 to 10 dpa. *GaSus4* was expressed very slightly in stem, leaf and petal, and had a similar, but significant lower expression tendency in fibers at different developmental stages compared to *GaSus3*. The highest transcript levels for *GaSus5* was also detected in fiber at 15 dpa, while very low expression levels for this gene was observed in fibers around at 0 and 5 dpa, as well as in root, leaf and petal*.* In addition, transcripts in fibers were continuously increasing with cotton fiber development from 5 to 15 dpa, but declining observably at 20 dpa. The expression pattern of the other two paralogues, *GaSus6* and *GaSus7*, were highly divergent. *GaSus6* was expressed in all tissues examined, but with relatively low levels in fibers. In contrast, *GaSus7* showed distinct tissue-specific patterns with its transcripts were only detectable in two tissues: very weak in stems and much abundant in petals.

As it has been documented repeatedly that the activity of Sus was highly correlated to cotton seed and fiber growth and development, the relative transcription level of five fiber-related *GaSus* genes were therefore quantitatively compared in fiber cells at different developmental stages. As shown in Figure 6b, at fiber initiation and early elongation stages (0–10 dpa), *GaSus3* was the gene that had significantly higher expression levels than other paralogues in cotton Sus family. As the fiber develops further from 10 to 15 dpa, *GaSus5* and *GaSus1* were becoming the predominant forms, contributing approximately 70% of the total amount of Sus transcripts in fiber cells. However, with the fiber develop further from 15 to 20 dpa, the dominant position of *GaSus5* was lost and replaced by *GaSus1* and *GaSus3*.

## Discussion

Recently, benefiting from the whole genome sequencing of model plants, dozens of genes encoding Sus isozymes have been identified from various plant species using the comparative genome approaches. Typically, the complete genomic sequence of the model species *Arabidopsis* and rice allows revealing a total of six Sus genes in each of the two plants, representing the entire Sus gene members of two groups of flowering plants, dicots and monocots. Although the exact number of Sus genes in most of other plants could not be reliably evaluated due to the incomplete coverage of genome sequencing, cotton, however, appears to have more distinct Sus genes than *Arabidopsis* and rice. Previously four distinct genes encoding different types of cotton Sus isoforms, *SusA* (*GhSus3*, accession no. U73588), *SusBSusC* and *SusD*, had been identified in the tetraploid upland cotton species [44]. Our present work through molecular cloning and database searching brings the number of presently known members of cotton Sus gene family to at least seven, comprising one of the largest Sus gene families described thus far. Given the limited knowledge of cotton Sus gene family for us to data, further investigation of their evolutionary relationships, molecular structures, as well as their expression patterns therefore becomes an important step towards comprehensive understanding their molecular mechanism and possible functions involved in different growth processes in cotton.

### Evolutionary conservation and divergence of cotton Sus genes

Although the evolution of multigene families involves multiple mechanisms, comprehensive analysis of phylogenetic tree and exon/intron gene structures, to a certain extent, allow us to make some generalizations and predictions about the possible origin of and relationships between different isoforms of Sus, as well as their possible function. Plant Sus proteins have been historically divided into at least three major groups (Sus1, SusA and New Group/NG) on the basis of phylogenetic tree and molecular structures analysis of their sequences [24,29]. Phylogenetic analysis of cotton *GaSus* genes and other plant homologues in our work corroborated this classification (for unification and simplification, in this study, we renamed them as Sus I, II and III, respectively), and further support the idea that higher plant species may have at least one gene for each of the three groups [24]. The presence of five cotton Sus genes, *GaSus1* to *5*, in the Sus I group that cluster together with other dicot genes and separately from multiple monocot Sus genes is the direct evidence of that multiple gene duplication events occurred after the monocot/dicot divergence both within dicots and monocots, giving rise to two subclades: dicot-specific Sus I group and the monocot-specific Sus I group. As the five cotton genes group together to the exclusion of citrus, *Arabidopsis* and other dicot homologues in our phylogenetic tree, it is suggestive of that gene duplication events that gave rise to the five cotton Sus genes occurred after the separation of cotton/citrus/*Arabidopsis* from a common ancestor. The presence of one cotton Sus gene in each of the other two groups, Sus II and III, with members from both dicot and monocot species, indicating that Sus II and III groups containing *GaSus6* and *GaSus7* are evolutionarily older than dicot Sus I group containing *GaSus1* to *5*.

It has been well demonstrated in many duplicated gene families that exon structure in homologues genes may even be conserved despite low sequence conservation [45]. Differences in exon/intron structures, created by intron deletions, insertions or both events, between paralogues therefore could be used, to some extent, to estimate the evolutionary history of gene families [46]. In the present work, comparative screening of introns in homologous genes indeed revealed that the number and position of introns are highly conserved among Sus genes in cotton and other more distantly related monocot plant species (Figure 2a, b and Figure 3). As the majority of Sus sequences in the three Sus groups contain 14 introns, in conserved positions, it is tempting to speculate that the gene duplications to arise three progenitors of Sus genes, each containing 14 introns in conserved positions, occurred early in the evolution of angiosperms and predated the divergence of monocots and dicots as many of the other gene families [47]. Interestingly, although the evolution of intron is considered to be under a low selection pressure compared to exon sequences, it seems that Sus genes in monocots tend to retain more introns than in dicots, such a case occurred particularly apparently in the Sus I group, in which members from dicot cotton and *Arabidopsis*, without exception, have lost introns 5 and 12. Since that members from other dicots, such as *Leguminosae Lotus japonicus* and *Rutaceae* citrus, of this group also have lost the two introns [24,29], it thus led to the inference that at least twice rounds of intron loss events have occurred in the evolution of the dicot Sus genes related to Sus I group including the five cotton Sus paralogues (*GaSus1* to *5*), one relatively earlier event of concerted intron loss of introns 5 and 12 occurred immediately after the dicot/monocot split but predated the separation of cotton and most of the other dicots. After this event, independent events of intron loss of introns 2, 4, 6, 11 and 13 occurred related to different dicots members in Sus I group. A comparison of the number and density of introns in the gene structures of the five cotton paralogues belong to the Sus I group further demonstrated that *GaSus5* was produced from relatively more ancient duplications in comparison with its three paralogues *GaSus1GaSus3* and *GaSus4.* Intriguingly, although Sus II group is thought to be evolutionarily older than dicot Sus I group, members in Sus II group appear to have underwent a relatively slower evolutionary rate, since that exon/intron structures of dicot genes (including cotton *GaSus6*) in this group have greater similarity to those of monocot Sus genes and also to the putative ancestral Sus genes (Figure 3). The most remarkable feature of the Sus III group is that members, whether from dicots or monocots, without exception, exhibit a 3' extension resulting in either a longer (*AtSus6OsSus6* and *GaSus7*) or additional exons (*AtSus5LjSus6* and *OsSus5*) in their 3' regions, it is tempting to speculate that a sequence with at least two introns had been introduced to the 3' region of the progenitor of Sus III group probably by an ectopic recombination that occurred before the monocots/dicots splits. The absence of commonalities on sequence and exon/intron structure in the extended regions among different members may be due to that the extended region did not involve in the definition of the protein activity of Sus (Figure 1) and thus suffered a low selection pressure during evolution. As the cotton *GaSus7* have an exon/intron structure similar to the *Arabidopsis AtSus5* (except *AtSus5* have an additional intron and exon in its extended 3' region), confirming that *AtSus5*, but not *AtSus6*, is the orthologuous gene of cotton *GaSus7*. This is also indicative of that the loss of intron 1, 6 and 12 from cotton *GaSus7* were the events that occurred before the separation of cotton and *Arabidopsis* lineages.

Synthesizing what we have learned from the phylogenetic tree analysis and exon/intron structure comparison, we proposed the following sequence of events to account for the evolution of the cotton Sus gene family. The first relatively earlier event that occurred before the split of monocots and dicots led to the duplication of an ancestral gene containing 14 introns to progenitors of *Sus6* and *Sus7.* A second duplication that occurred also predated the monocots/dicots split was associated with the arising of the precursor of *Sus1* to *5*. Following the divergence from a common ancestor of citrus and *Arabidopsis*, a duplication occurred in cotton lineages and giving rise to *Sus5*, which subsequently underwent independent evolution and retained as a single gene, while its duplicate has expanded into four paralogues (*Sus1* to *4*), among which, *GaSus3* and *GaSus4* were produced from the most recent duplication events and consequently became the closest related genes in cotton Sus family, their closeness is well reflected in the terminal subgroups of the phylogenetic tree (Figure 4). Therefore, it might be unsurprising that *GaSus4* would have a tissue-expression pattern or physiological functions similar to that of *GaSus3* in cotton. *GaSus2*, however, seemingly have underwent a more rapid evolutionary rate and thus have a relatively longer separating branch and lower degree of sequence identity compared with the other three paralogues. It's interesting to note that there are only two *Arabidopsis* genes in the Sus I group, although *Arabidopsis* lineage had underwent one round of whole genome duplication since it split from a common ancestor shared with cotton [48]. The discrepancy of Sus gene numbers in related species well supports the theory that genome duplication as well as the following gene loss (diploidization) are prevalent features of plant genomes [49].

It should be emphasized that, although our efforts in this study, though molecular cloning, massive sequencing and database hunting, have brought the cotton Sus gene family to be one of the largest Sus family described to date, we could not completely exclude other paralogues for each of the three groups which might be present in the cotton species. The presence of only one cotton Sus member, compared with both *Arabidopsis* and *Lotus japonicus* having two members, in each of the Sus II and III groups in our phylogenetic tree also implies the possibility of additional, unidentified cotton Sus paralogues existent within the two groups. The topology of the phylogenetic tree in future might have some deviation in the order of evolutionary distances for different members of cotton Sus family. Therefore, more information, especially the chromosomal distribution of these cotton Sus genes, is needed to determine a more precise evolutionary relatedness among these cotton Sus genes. Although this study focused mainly on Sus genes in cotton species, the examined relative high conservation of the orthologous genes of Sus family in different cotton interspecies may lend strong evidence to support the further comparative genomics analysis across the entire family of Malvaceae.

### Functional divergence of the cotton GaSus gene family

It has been well recognized that gene duplication followed by functional diversity (evolutionary changes in expression patterns and/or protein property) has played a crucial role in driving evolutionary novelty that allow organisms to differentiate new organs or increase fitness to new environments [50,51]. To data, although the functions of a few individual isoforms of Sus have been characterized in tetraploid cotton plants [52], there is no systematic functional analysis of expression patterns for different groups of diploid cotton Sus gene family.

In this study, we demonstrated that differential expression of the *Sus* genes occurs in cotton, as did the members of this Sus family in several other plant species, such as *Arabidopsis*[20,23], rice [24], *Lotus japonicus*[25], citrus [29], and Bamboo [37]. The differential expression profiles may suggest that Sus is implicated in a range of physiological processes in the cotton plant. The specialized expressions of Sus genes may also reflect the divergent evolution of Sus gene regulatory elements that are required for controlling the development of particular cells or tissue types during cotton plant growth [53].

Previous studies, based on the analysis of mutant and/or transgenic plants with reduced Sus activity, have demonstrated that cotton fiber and seed development are significantly correlative to the changes of Sus activity in ovule epidermis and endosperm [34,54]. The transcription data presented here indeed provide direct evidence for strong expression of some *GaSus* genes in cotton ovules and fibers (Figures 5,6a,b). The relatively high expression level of Sus genes coincident with the initiation and elongation of the fiber cell well supports the standpoint that Sus in cotton plays a pivotal role in cotton fiber growth and development. Apparently, development-dependent regulation on Sus genes, involved in cell initiation and elongation during cotton ovule and fiber development had evolved. This is typically shown by the expression of *GaSus3* and *GaSus5*. Transcriptional analysis revealed that the *GaSus3* had significantly higher expression levels in 0 dpa ovules and early developing fibers compared to the other paralogues in cotton, and displayed gradually decreasing expression levels in fibers from 0 to 10 dpa, suggesting that *GaSus3* may play a major role in the early stages of fiber development, particularly in fiber initiation and early elongation. The hypothesis is indirectly supported by the previous study that suppression of the expression of *GhSus3* (U73588), an orthologous gene of *GaSus3*, in tetraploid upland cotton, repressed cotton early seed development, fiber cell initiation and elongation. In contrast to *GaSus3* expression, the transcripts of *GaSus5* were very low at 0 and 5 dpa, but increased continuously in abundance and reach its highest level in fibers around at 15 dpa, and then a significant decrease as the fiber cells developed further, suggesting that *GaSus5* may primarily participated in fiber cell elongation and primary cell wall synthesis. The differential, complementary expression profiles of *GaSus3* and *GaSus5* is the direct evidence of that specialized *GaSus* genes might have been evolved to meet the requirement of the carbohydrates for cellulose synthesis, fiber cell initiation and elongation during cotton fiber development. Interestingly to note, of the seven cotton Sus genes, *GaSus7* is not only the oldest duplicated gene, but also is the only one which is transcriptionally active, but not expressed in ovule and fiber cells. The strong transcription level of *GaSus7* in petal implies that *GaSus7* may be primarily involved in the regulation of floral organs, but not the ovules and fibers development in cotton. As *GaSus6,* another old duplicated gene, is also lowly activated in cotton fiber cells compared to those relatively recent duplicated paralogues, such as *GaSus1* and *GaSus3* (Figures 6a,b), it is tempting to speculate that the recent duplications of Sus genes may be important or highly correlate to the cotton fiber cell differentiate. 7This might also be one of the reasonable explanations why cotton could retain more duplicated genes in the Sus I group than most of the other phylogenetic related species, such as *Arabidopsis*.

**Figure 7:**
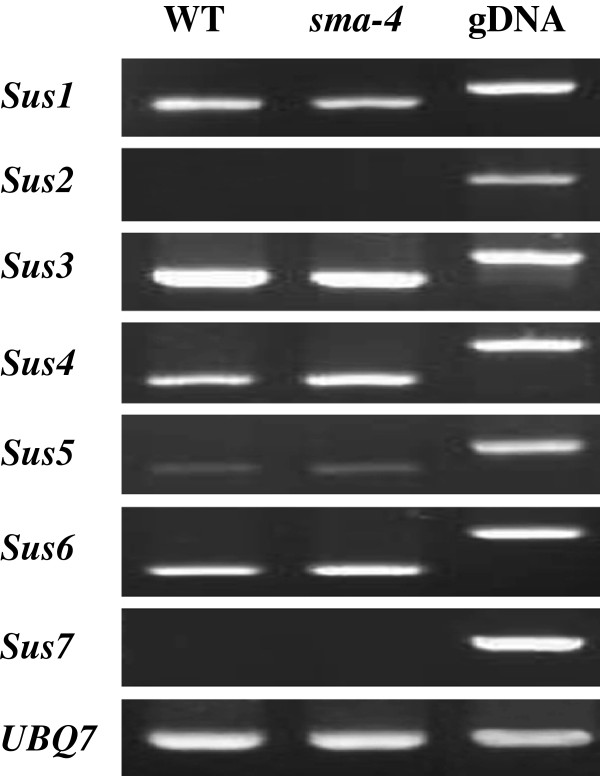
**Transcriptional analysis of the seven *GaSus *genes in ovule at 0 dpa in wide-type and fiberless mutant (*sma-4*) cotton plants.** PCR products amplified from genomic DNA (g) containing at least one introns were taken as controls to exclude any DNA contamination from the cDNA templates.

Previous studies have demonstrated that Sus expression was much heterogeneity among cotton ovule epidermal cells [54,55]. Seemingly, only those with high levels of Sus transcripts could actually differentiate into long fibers, while cells with lower or undetectable levels exhibited smaller or even no initiation out of the ovule epidermis. Transcriptional analysis of a fiberless cotton mutant *sma-4*[56] in this study, however, did not show significant difference of mRNA levels in ovules at 0 dpa for all the seven Sus genes compared to the wild-type plants (Figure 7). This finding was contradictory to the earlier study on another fiberless cotton mutant *fls*, which had no signal for Sus mRNA and protein in the ovule epidermal cells at 0 dpa, while the wide type showed strong Sus mRNA signals [54]. Such results suggested that the regulatory network leading to cotton fiber growth and development could well be more subtle and complex, and multiple genes, upstream/downstream of the Sus genes, might also have been evolved into strict controlling of cotton fiber cell initiation and elongation [57].

It is worth noting that although *GaSus3* was ubiquitously expressed in the cotton plants, knock down of the *Sus3* expression in upland cotton virtually unaffected vegetative growth and flowering in most of the transgenic cotton plants [34], suggesting possible functional redundancies within the cotton Sus gene family. This might be true as a result of that similar or overlapping expression pattern to the *GaSus3* genes were also observed in the case of *GaSus4*, as well as in other paralogues in cotton. Similar results and conclusion were also obtained from the study on *Arabidopsis* Sus mutants that lacking individual isoforms of Sus failed to cause obvious growth phenotypes compared to the wide type of *Arabidopsis* plants [20], suggesting that redundancy within Sus gene family would ensure that sucrose metabolisms in particular cells or organs will be relatively insensitive to mutations and evolutionary robust.

One of the most common fate of gene duplications is thought to be elimination of one of the duplicate pair [58]. The undetectable expression of *GaSus2* in all the tissues examined and its relatively faster evolutionary rate compared to the other paralogues led to the suggestion that *GaSus2* may be a pseudogene or is on the way to become a pseudogene. The loss of some peptides including the conserved, functional Ser phosphorylation site (Figure 1) in the N-terminal region of the GaSus2 deduced polypeptides is also the indirect evidence that supports the hypothesis. Similarly in rice, a putative Sus gene fragment identified in the genome database searches, is also inactive and probably as a consequence of the loss of some exons and inclusion of multiple stop codons within its putative coding region [24]. Such cases were also found in some other multiple gene families, for example, the *Arabidopsis* actin gene family contains 10 distinct members, of which eight are functional genes and two are pseudogenes [59,60].

## Conclusions

This study provides the first comprehensive evaluation of the evolutionary genesis, phylogenetic relationships, exon/intron structure organization, and tissue-expression pattern of each member of the Sus gene family in cotton species. The results presented here could offer an useful foundation and framework for future research work on understanding the potential physiological roles for each cotton Sus gene and the evolution of Sus gene family in response to sucrose metabolism during cotton fiber development, as well as the structure-function relatedness between the members of cotton Sus gene family. We also realize that the different Sus genes in cotton might play different roles in regulating sucrose metabolism and fiber development, as well as possible functional interactions or even redundancies. Such functional expectations need to be experimentally verified by characterizing the spatio-temporal expression dissection, Sus enzyme activity assay and knockout/knockdown mutants of these Sus genes in the near future.

## Methods

### Plant material and growth conditions

*G. arboreum* (acc. A_2_-47), which was obtained from USDA collection through Dr. Peng Chee (University of Georgia, UGA), was used in this study for cloning Sus genes and gene expression analysis. The seeds were delinted, germinated and maintained in pot culture under natural conditions for three weeks, and then the plantlets were either harvested for collecting the roots, stems and leaves samples or transplanted to open field for continuing growing. For analysis of Sus gene expression during cotton fiber development, each flower was tagged on the day of flowering and were taken as 0 dpa (days post anthesis). Developing ovules were subsequently harvested at various developmental stages from 0 to 20 days after flowering, and fibers were carefully scraped from the epidermis of the ovules. The collected samples were immediately frozen in liquid nitrogen and stored at −80°C for subsequent RNA isolation.

Except *G. arboreum*, DNAs used for gene structure deduction and phylogenetic analysis were from *G. anomalum* (B1) and *G. sturtianum* (C1) obtained from Dr Xinlian Shen (Jiangsu Academy of Agricultural Sciences, China), and *G. raimondii* (D5) and *Gossyploides kirkii* (K) provided kindly by Dr. Andrew Paterson (UGA), here *Gossypioides kirkii* was used as an our group species.

### Cloning of Sus genes in diploid cotton of *G. arboreum*

Based on the sequences of plant sucrose synthase genes accessioned in GenBank, we designed multiple pairs of degenerate primers from the conserved regions for using to amplify the homologs in diploid fiber cotton (*G. arboreum*). The PCR products obtained from both cDNA and genomic DNA amplification were cloned into the pMD19-T cloning vector (TaKaRa Biotechnology, Dalian, China), and then transformed into *E.coli* cells (DH5α) for massive sequencing. The obtained sequences were submitted to NCBI database for blast analysis. In addition, the cotton EST database were also searched using *Arabidopsis* Sus genes as queries to identify putative unidentified members belong to the cotton Sus gene family.

The full-length cDNAs of the cotton Sus genes were obtained using the RLM-RACE approaches (FirstChoiceRLM-RACE Kit, Ambion, USA). Approximately one and 10 μg high-quality total RNA were used respectively for the 3′ and 5′ RLM-RACE protocols, strictly following the manufacturer’s instructions.

### Phylogenetic and gene structure analysis

The sequence data used in this study were collected using the keyword “sucrose synthase” and a query search in the GenBank using the known Sus gene sequences from cotton and *Arabidopsis*. Multiple alignment of the nucleotide and deduced amino acid sequences were performed using the programs ClustalX (version 1.8) [61], and DNAMAN (version 6.0)( http://www.lynnon.com/) with default gap penalties. A phylogenetic tree was constructed from amino acid sequences of deduced Sus proteins by neighbor-joining algorithms wrapped in the MEGA 5.0 phylogeny program ( http://www.megasoftware.net).

Exon/intron structures analysis of the target Sus genes were either conducted by comparing the cDNA sequences and their genomic DNA sequences or predicted online using the FGENESH algorithm ( http://linux1.softberry.com/berry.phtml?topic=fgenesh&group=programs&subgroup=gfind). Predicted conserved domains were screened within the deduced amino acid sequences of corresponding Sus genes using the interproscan algorithm web server ( http://www.ebi.ac.uk/Tools/pfa/iprscan/).

### DNA and RNA extraction

Genomic DNA was isolated from young cotton leaves using a CTAB method as described previously [62]. Total RNA was isolated from various tissue samples using the CTAB-sour phenol extraction method as described by Jiang *et al*., [63]. RNA samples were treated with DNase I (TaKaRa) after the extraction to eliminate the trace contaminants of genomic DNA.

### cDNA preparation and semiquantitative RT-PCR analysis

For conducting reverse transcription (RT)-PCR analysis, approximately two micrograms of DNA-free total RNA from each sample was used to synthesize first-strand cDNA in a 20-μl reaction solution using a M-MLV reverse transcription kit (TaKaRa), and the synthesized cDNAs were used as templates in the following RT-PCR reactions.

For each target Sus gene, PCR amplification was performed using Takara Taq polymerase with specific primer pairs listed in Additional File 4. The PCR was conducted in a heated-lid thermalcycler (Eppendorf, Germany) by following procedure: pre-denaturation at 95°C for 3 min, followed by 28–30 cycles of 30 s at 94°C, 60 s at a specific annealing temperature at 52-55°C for each gene, and 90 s at 72°C. A cotton constitutive gene, *UBQ7*, was used as an internal standard to adjust the relative quantity of the cDNA of each sample used in the RT-PCR analysis.

To confirm the specificity of each primer and also to exclude any genomic contamination in PCR amplification, each of the target genes was also amplified with the same PCR procedures from the genomic DNA, which contains at least one intron in its amplified products. The amplified fragments were examined by electrophoresis on a 1.5-2.0% (w/v) agarose gel and visualized by ethidium bromide (EB) staining.

### Quantitative real-time RT-PCR (qRT-PCR)

Real-time quantitative RT-PCR was performed to relatively quantify the transcription levels of cotton Sus genes expressed in fibers at different developmental stages. The cDNAs used for detecting gene expression were the same as those used for semiquantitative RT-PCR analysis. The reaction was conducted on the Applied Biosystems 7300 Real-Time PCR System using the SYBER premix ExTaq kit (TaKaRa) according to the Manufacturer's instructions. The amplification of the target Sus genes was monitored by SYBR-Green fluorescence signal every cycle. The Ct (cycle threshold) value, which was defined as the PCR cycle at which a significant increase of reporter florescence signal is detected instantly, was used as a measure for the starting copy numbers of the target Sus gene. Relative quantitation for expression level of each *GaSus* gene was standardized to the expression level of the cotton constitutive *UBQ7* gene, calculated by the formula Y = 10^-(ΔCt/3)^ (ΔCt is the differences of Ct between the target Sus and the control UBQ7 products; ΔCt = Ct_GaSus_ - Ct_UBQ_). The specificity of primers designed for real-time RT-PCR (Table 2) was confirmed by running products on agarose gels and by sequencing after the PCR reaction. The detailed protocol of the quantitative analyses was described by Li *et al*., [60].

**Table 2:** Gene-specific primers used for real-time RT-PCR amplification

**Gene**	**Forward primer**	**Reverse primer**
*GaSus1*	acgggttctggaagcatgtgtc	ccccggcaacttcaatttcaat
*GaSus3*	cgccgtgagagtcgtcgttacc	ccaagaaaaaccggcccaatg
*GaSus4*	gccgccgttacctggagatgt	cccgcctcttcctttgttttac
*GaSus5*	cggccaatacgagagtcacatc	cggcttgttgcggtcttttag
*GaSus6*	ggctgatgacattggctggagta	cgcaatcaaccagacccttaaat
*GaSus7*	cgcgtttcgacatttatccttatc	tgcgcaatcgtagcctgtgtt
*UBQ7*	gaaggcattccacctgaccaac	cttgaccttcttcttcttgtgcttg

## Abbreviations

Sus: Sucrose synthase; EST: Expressed sequence tag; dpa: Days post anthesis.

## Competing interests

The authors declare that they have no competing interests.

## Authors’ contributions

AQC and JKR contributed to the experimental design and manuscript drafting. QPL contributed to the manuscript editing. AQC, SEH and ZL performed the primer design, Sus gene cloning and RT-PCR validation. FFL, MQD and QPL performed the RNA extraction and bioinformatics analysis. All authors have read and approved the final manuscript.

## Supplementary Material

Additional file 1 Amino acid sequences analysis of the cotton Sus genes. Click here for file

Additional file 2 **Multiple alignment of the amino acid sequences between three Sus genes *GaSus1, GaSus3 *and *GaSus4.*** Click here for file

Additional file 3 **Multiple alignment of the DNA sequence between five orthologs of Sus7 cloned from four diploid cotton species *G. arboreum *(A2, *GaSus7*), *G. anomalum *(B1**, ***GbSus7*), *G. sturtianum *(C1**, ***GcSus7*) and *G. raimondii *(D5, *GdSus7*) and one outgroup species, *Gossypioides kirkii *(K, *GkSus7*).** Corresponding regions shaded in colours represent sequence identities between these orthologous genes. The exon sequence is shown by single underline, and the intron sequences are shown by double underlines. The arrows upon the first intron sequence indicate the unusual GC/AG splicing sites in the *Sus7* genes. The SSRs (simple sequence repeats; microsatellites) in the five orthologs are boxed and shaded. Click here for file

Additional file 4 **Gene-specific primers used for semi-quantitative RT-PCR amplification.** The numbers out and in parentheses represent expected size of PCR products amplified from cDNA and genomic DNA, respectively. Click here for file
