# Supplementary material for: Analyses of the sucrose synthase gene family in cotton: structure, phylogeny and expression patterns
Source: BMC Plant Biol. 2012 Jun 13;12:85. doi: 10.1186/1471-2229-12-85 (PMC3505178; doi:10.1186/1471-2229-12-85)
Supplement: Additional file 1 — Amino acid sequences analysis of the cotton Sus genes. [file 1471-2229-12-85-S1.pdf]

Additional file 1. Amino acid sequences analysis of the Sus genes from five cotton species.

Sus proteins	Amino acid size	Molecular weight(kDa)	Isoelectrical point
GaSus1	806	92.80	6.52
GaSus2	798	91.75	6.58
GaSus3	805	92.66	6.54
GaSus4	806	92.61	6.67
GaSus5	796	90.26	6.26
GaSus6	809	92.23	6.39
GaSus7	824	93.14	7.27
GbSus1	806	92.77	6.52
GbSus2	798	91.96	6.46
GbSus3	805	92.69	6.54
GbSus4	806	92.51	6.40
GbSus5	796	90.54	6.83
GbSus6	809	92.10	6.39
GbSus7	824	93.20	7.31
GcSus1	806	92.82	6.43
GcSus2	800	92.23	6.63
GcSus3	805	92.69	6.38
GcSus4	806	92.42	6.40
GcSus5	796	90.47	6.40
GcSus6	809	92.14	6.33
GcSus7	824	93.07	7.26
GdSus1	806	92.76	6.58
GdSus2	798	92.18	6.55
GdSus3	805	92.66	6.52
GdSus4	806	92.61	6.46
GdSus5	796	90.34	6.43
GdSus6	809	92.30	6.33
GdSus7	824	93.10	7.07
GkSus1	806	92.62	6.36
GkSus2	791	90.91	6.10
GkSus3	805	92.66	6.54
GkSus4 (Partial)	-	-	-
GkSus5	796	90.44	6.20
GkSus6	809	92.11	6.29
GkSus7	824	93.52	6.84
