# Supplementary material for: Analyses of the sucrose synthase gene family in cotton: structure, phylogeny and expression patterns
Source: BMC Plant Biol. 2012 Jun 13;12:85. doi: 10.1186/1471-2229-12-85 (PMC3505178; doi:10.1186/1471-2229-12-85)
Supplement: Additional file 2 — Multiple alignment of the amino acid sequences between three Sus genes GaSus1, GaSus3 and GaSus4. [file 1471-2229-12-85-S2.pdf]

Additional file 2. Multiple alignment of the amino acid sequences between three Sus genes *GaSus1*, *GaSus3* and *GaSus4*.

```
GaSUS1     MAERALTRVHSLRERLDSTLTAHRNEILALLSRIEGKGKGILLHHQIILEFEAIPEENRKKLADGAFFEILKASQEAIVLPPWVALAVRPRPGVWEYIRVNVHALVVEEL     110
GaSUS3     MADRVITRVHSLRERLDETLLAHRNEILALLSRIEGKGKGILQHHQIILEFEAIPEENRKKLANGAFFEVLKASQEAIVLPPWVALAVRPRPGVWEYIRVNVHALVVEEL     110
GaSUS4     MAERVITRVHSLRERLDDTLIAHRNEVLALLTRIEGKGKGILQHHQIILEFEAIPEETRKKLADGAFSEILRASQEAIVLPPWVALAVRPRPGVWEYIKVNVHALVVEEL     110
Consensusma r  trvhslrerld tl ahrne lall riegkgkgil hhqiilefeaipee rkkla gaf e l asqeaivlppwvalavrprpgvweyi vnvhalvveel

GaSUS1     TVAEYLRFKEELVDGSSNANFVLELDFEPFNASFPRPTLSKSIGNGVEFLNRHLSAKLFHDKESMHPLLEFLKVHCHKGKNMMLNDRIQNLNSLQHVLRKAEEYLVALPA     220
GaSUS3     TVAEYLHFKEELVDGSSNGNFVLELDFEPFNSSFPRPTLSKSIGNGVEFLNRHLSAKLFHDKESMHPLLEFLRVHCHKGKNMMLNDRIQNLNALQHVLRKAEEYLGTLPP     220
GaSUS4     TVAEYLHFKEELVDGSANGNFVLELDFEPFNASFPRSTLSKSIGNGVEFLNRHLSAKLFHDKESMHPLLEFLKVHCHKGKNMMLNDRIQNLNSLQYVLRKAEEYLGTLPA     220
Consensustvaeyl fkeelvdgs n nfvleldfepfn sfpr tlksigngveflnrhlsaklfhdkesmhpllefl vhchkgknmmlndriqnln lq vlrkaeeyl  lp

GaSUS1     ETPYAEFEHKFQEIGLERGWGDTAERVLEMIQLLLDLLEAPDPCTLEKFLGRIPMVFNVVILTPHGYFAQDNVLGYPDTGGQVVYILDQVRALENEMLNRIKQQGLNITP     330
GaSUS3     ETPCAEFEHRFQEIGLERGWGDTAERVLEMIQLLLDLLEAPDPCTLEKFLGRIPMVFNVVILTPHGYFAQDNVLGYPDTGGQVVYILDQVRALENEMLLRIKQQGLNITP     330
GaSUS4     ETPYTELEHKFQEIGLERGWGDTAGRVLEMIQLLLDLLEAPDPCTLEKFLGRVPMVFNVVILTPHGYFAQDNVLGYPDTGGQVVYILDQVRALENEMLLRIKQQGLNITP     330
Consensusetp  e eh fqeiglergwgdta rvlemiqllldlleapdpctlekflgr pmvfnvviltphgyfaqdnvlgypdtggqvvyildqvraleneml rikqqglnitp

GaSUS1     RILIITRLLPDAVGTTCGQRLEKVYGTEYSDILRIPFRTEKGIVRRWISRFEVWPYLETYTEDVAHEISKELQGKPDLIIGNYSDGNIVASLLAHKLGVTQCTIAHALEK     440
GaSUS3     RILIISRLLPDAVGTTCGQRLEKVYGTEHSDILRVPFRTEKGIVRKWISRFEVWPYLETYTEDVAHEISKELQGKPDLIIGNYSDGNIVASLLAHKLGVTQCTIAHALEK     440
GaSUS4     RILIITRLLPDAVGTTCGQRVEKVYGTEYSDILRVPFRTEKGIVRRWISRFEVWPYLETYTEDVAHEISKELQGKPDLIIGNYSDGNIVASLLAHKLGVTQCTIAHALEK     440
Consensusrilii rllpdavgttcggr ekvygte sdilr pfrtekgivr wisrfevwpyletytedvaheiskelqgkpdliignysdgnivasllahklgvtqctiahalek

GaSUS1     TKYPDSDIYWKKLEDKYHFSCQFTADLFAMNHTDFIITSTFQEIAGSKDTVGQYESHTAFTLPGLYRVVHGIDVFDPKFNIVSPGADMSIYYPYTEEKKRLKHFHSEIEE     550
GaSUS3     TKYPDSDIYWKKLEDKYHFSCQFTADLFAMNHTDFIITSTFQEIAGSKDTVGQYESHTAFTLPGLYRVVHGIDVFDPKFNIVSPGADMEIYFPYTEEKRRLKHFHPEIED     550
GaSUS4     TKYPDSDIYWKKLEDKYHFSCQFTADLIAMNHTDFIITSTFQEIAGSKDTVGQYESHTAFTLPGLYRVVHGIDVFDPKFNIVSPGADMSIYFPYTEKKRRLKHFHPEIED     550
Consensustkypdsdiywkkledkyhfscqftadl amnhtdfiitstfqeiagskdtvgqyeshtaftlpglyrvvhgidvfdpkfnivspgadm iy pyte k rlkhfh eie

GaSUS1     LLYSKVENEEHWCVLNDRNKPILFTMARLDRVKNLTGLVEWYGKNAKLRELVNLVVVGGDRRKESKDLEEKAEMKKMFELIEKYKLNGQFRWISSQMNRVRNGELYRYIC     660
GaSUS3     LLYSKVENEEHLCVLNDRNKPILFTMARLDRVKNLTGLVEWYGKNAKLRELANLVVVGGDRRKESKDLEEKAEMKKMFELIDKYNLNGQFRWISSQMNRIRNGELYRYIC     660
GaSUS4     LLYSKVENEEHLCVLNDRNKPILFTMARLDRVKNLTGLVEWYGKNAKLRELVNLVVVGGDRRKESKDLEEKAEMKKMFELIKTYKLNGQFRWISSQMNRVRNGELYRYIC     660
Consensusllyskveneeh cvlndrnkpilftmarldrvknltglvewygknaklrel nlvvvggdrrkeskdleekaemkkmfeli  y lngqfrwissqmnr rngelyryic

GaSUS1     DTKGAFVQPALYEAFGLTVVEAMTCGLPTFATCNGGPAEIIVHGKSGFNIDPYHGDQAAEILVDFFEKCKTDPSYWTKISEGGLKRIEEKYTWKIYSERLLTLTGVYGFW     770
GaSUS3     DTKGAFVQPALYEAFGLTVVEAMTCGLPTFATCNGGPAEIIVHGKSGFNIDPYHGDQAADILVDFFEKCKKDPSHWDKISQGGLKRIEEKYTWKIYSERLLTLTGVYGFW     770
GaSUS4     DTKGAFVQPALYEAFGLTVVEAMTCGLPTFATCKGGPAEIIVHGKSGFNIDPYHGDQAAEILVDFFDKCKKEPSHWNDISEGGLKRIQEKYTWQIYSERLLTLTGVYGFW     770
Consensusdtkgafvqpalyeafgltvveamtcglptfatc ggpaeiivhgksgfnidpyhgdqaa ilvdff kck  ps w  is gglkri ekytw iyserlltltgvygfw

GaSUS1     KHVSNLDRLESRRYLEMFYALKYRKLAESVPLAVEE                                                                                806
GaSUS3     KHVSNLERRESRRYLEMFYALKYRKLAESVPLA.EE                                                                                805
GaSUS4     KHVSNLDRRESRRYLEMFYALKYRKLAESVPLAEEE                                                                                806
Consensuskhvsnl r esrrylemfyalkyrklaesvpla ee
```
