# Supplementary material for: Analyses of the sucrose synthase gene family in cotton: structure, phylogeny and expression patterns
Source: BMC Plant Biol. 2012 Jun 13;12:85. doi: 10.1186/1471-2229-12-85 (PMC3505178; doi:10.1186/1471-2229-12-85)

Additional file 3. Multiple alignment of the partial DNA sequence between five orthologs of Sus7 cloned from four diploid cotton species *G. arboreum* (A2, *GaSus7*), *G. anomalum* (B1, *GbSus7*), *G. sturtianum* (C1, *GcSus7*) and *G. raimondii* (D5, *GdSus7*) and one outgroup species, *Gossypioides kirkii* (K, *GkSus7*). Corresponding regions shaded in colours represent sequence identities between these orthologous genes. The exon sequence is shown by single underline, and the intron sequences are shown by double underlines. The arrows upon the first intron sequence indicate the unusual GC/AG splicing sites in the *Sus7* genes. The SSRs (simple sequence repeats; microsatellites) in the five orthologs are boxed and shaded.

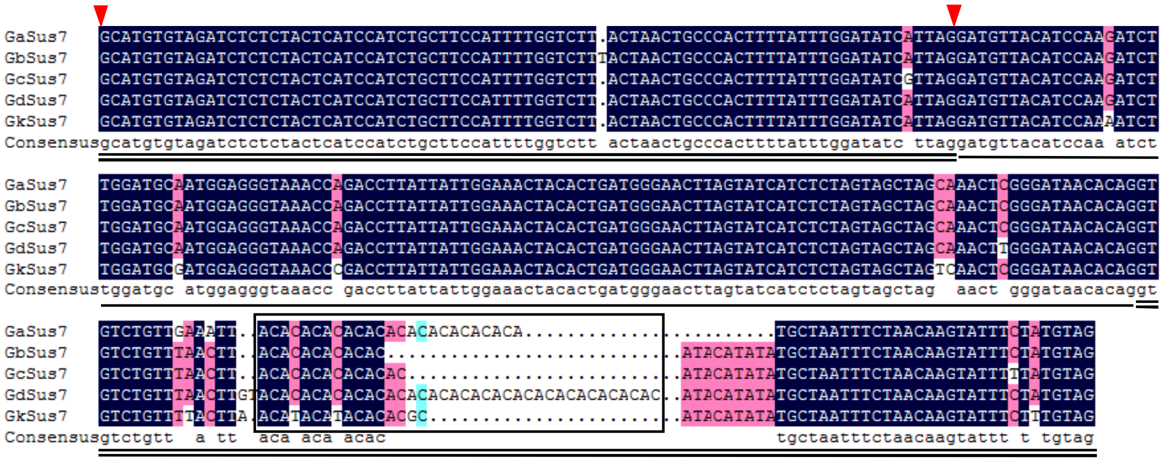

Supplement: Additional file 3 — Multiple alignment of the DNA sequence between five orthologs of Sus7 cloned from four diploid cotton species G. arboreum (A2, GaSus7), G. anomalum (B1, GbSus7), G. sturtianum (C1, GcSus7) and G. raimondii (D5, GdSus7) and one outgroup species, Gossypioides kirkii (K, GkSus7). Corresponding regions shaded in colours represent sequence identities between these orthologous genes. The exon sequence is shown by single underline, and the intron sequences are shown by double underlines. The arrows upon the first intron sequence indicate the unusual GC/AG splicing sites in the Sus7 genes. The SSRs (simple sequence repeats; microsatellites) in the five orthologs are boxed and shaded. [file 1471-2229-12-85-S3.pdf]
