# Supplementary material for: Analyses of the sucrose synthase gene family in cotton: structure, phylogeny and expression patterns
Source: BMC Plant Biol. 2012 Jun 13;12:85. doi: 10.1186/1471-2229-12-85 (PMC3505178; doi:10.1186/1471-2229-12-85)
Supplement: Additional file 4 — Gene-specific primers used for semi-quantitative RT-PCR amplification. The numbers out and in parentheses represent expected size of PCR products amplified from cDNA and genomic DNA, respectively. [file 1471-2229-12-85-S4.pdf]

Additional file 4. Gene-specific primers used for semi-quantitative RT-PCR amplification. The numbers out and in parentheses represent expected size of PCR products amplified from cDNA and genomic DNA, respectively.

Gene	Forward Primer	Reverse Primer	Expected size (bp)
GaSus1	cggtctctaccgtgttgttcacgga	tgcccaaaacatgaagagaaacaaat	1063 (1236)
GaSus2	tactggaaggagcttgaggat	tccaccattggagatttcatt	0 (1079)
GaSus3	ggtctctaccgtgttgtacatggt	caagaaaaaccggcccaatgttat	947 (1221)
GaSus4	gcaaaggtggaccagctgaaatcatt	ccggccataaatatgaaaacaaca	464 (666)
GaSus5	ataagtatcacttctcctgtcag	caaggggaaacccaaagttgaaatt	1185 (1369)
GaSus6	ggtggtcctgcagagattattgaaca	cgcaatcaaccagacccttaaat	405 (604)
GaSus7	gcccgaggaactaagtgcaacc	tcggcaatgcaacggtagagt	962 (1323)
UBQ7	gaaggcattccacctgaccaac	cttgaccttcttcttcttgtgcttg	198 (198)
